# Support Needs in Carers of People With Parkinson’s From Early to Later Stages: A Qualitative Study With 36 Carers in 11 European Countries

**DOI:** 10.1177/08919887231168404

**Published:** 2023-04-20

**Authors:** Rowena K. Merritt, Sarah Hotham, Anette Schrag

**Affiliations:** 1 Centre for Health Services Studies, 2240 University of Kent, Canterbury, UK; 2 Department of Clinical Neurosciences, Institute of Neurology, Royal Free Campus, 61554 University College London, London, UK

**Keywords:** Parkinson’s disease, carers, qualitative, information

## Abstract

**Background:**

Parkinson’s Disease (PD) is associated with considerable carer burden, but there has been little qualitative research on the support needs of carers of People with Parkinson’s (PwP).

**Methods:**

Semi-structured in-depth interviews with carers of PwP in 11 European countries.

**Results:**

Interviews with 36 carers of PwP were analysed. At the time of diagnosis, carers often felt that they had a role in helping get a diagnosis and then in dealing with the impact of the diagnosis on the family. Information on medication was seen as particularly important for carers, and many of the carers felt that their informational needs differed from that of the PwPs. Many of the carers also felt that they needed to be present at all appointments to request referrals or ask for medication changes. Carers of those in the later stages of the disease often reported feeling isolated and not having any time for themselves.

**Conclusions:**

The involvement of carers should be addressed more actively in the management of Parkinson’s.

## Introduction

Parkinson’s disease (PD) is a chronic, progressive disorder which is classically characterised by slowness, rigidity, tremor, and difficulties walking, and can also lead to a range of nonmotor symptoms, including cognitive impairment, psychiatric complications, urinary dysfunction and sleep disturbances. As the condition progresses, the symptoms increase, and it can become increasingly difficult to carry out everyday activities without help. Despite this, most individuals with PD live in their own homes cared for by a family member.^1^ Assistance with physical care including feeding, dressing, and washing increases as the disease progresses, with a reported mean number of care activities increasing from 11 per day in the earlier stages of Parkinson’s to 30 per day in the later stages.^2^

Quantitative studies have demonstrated considerable carer burden in the later stages of the disease, both physically, mentally, and socially, with psychiatric complications of PD being a particular contributor to carer burden.^3^ However, there has been little qualitative research on the carers’ own experience of support needs from the early to the later stages. Understanding their particular needs can provide the basis for meaningful intervention to support them. We here report the results of a qualitative study conducted with 36 carers of people with PD (PwP) in 11 European countries.

## Methods

The research study was conducted as part of the multi-stakeholder project titled “*My PD Journey*” by the European Parkinson’s Disease Association (EPDA), which was designed to remove the hurdles that prevent people with Parkinson’s from receiving timely and appropriate treatment and individualised care. Semi-structured interviews on the experiences of carers living and looking after a person with PD were conducted in Denmark, France, Germany, Hungary, Ireland, Italy, Netherlands, Slovenia, Spain, Sweden, and the UK. Principles of Grounded Theory were used to guide the sampling, data collection and data analysis.^4^

## Recruitment and Sampling

Participants were predominantly identified and recruited through national Parkinson’s Associations who asked for volunteers to take part in the study. Through this strategy, 27 of the total 36 participants were recruited. The remaining participants (n = 9) were identified through snowball sampling.

The Parkinson’s Associations each identified 8 to 10 potential carers for the interviews, and then participants were selected to try and include carers with representative and balanced characteristics on four main criteria from each country: 1) age; 2) gender; and 3) number of years being a carer for someone with Parkinson’s disease; and 4) dwelling in rural and urban areas.

Although the number of in-depth interviews to be conducted should not be predetermined in Grounded Theory and recruitment should continue until data saturation is reached,^5^ a number of three participants from each of the 11 countries was agreed before the study commenced due to financial constraints (although in two countries more carers were interviewed due to cost savings with the translation services). Nevertheless, data saturation was reached.

## Data Collection

The individual interviews were conducted face-to-face or over the telephone (if requested by the participant). All participants were interviewed once. All interviews were recorded. Participants were informed of the intention to record in the study information sheet and the recorder was placed in a position clearly visible to each participant. Verbal informed consent was sought and obtained from all participants before the interview commenced. The interviews were transcribed verbatim and translated into English (if applicable) for analysis. Ethics approval for the analysis of the data was obtained from the UCL Research Ethics Committee (Project ID 14295/001).

## Interview Guide

An interview discussion guide was developed by RM, with input from the EPDA and AS. Topics covered included recognising the first signs and symptoms of PD and feelings at this point; reaction to diagnosis; perception towards current treatments and services being received; satisfaction with past and current treatment plans and healthcare professionals engagement; and informational and support needs throughout the progression of the disease.

## Data Analysis

All interviews were transcribed verbatim. The transcripts used accepted procedures for indicating exclamations, pauses and emotion.^6^ Transcriptions were imported into NVivo.^7^ Analysis of the individual interviews used the constant comparative method. Two researchers conducted the analysis and coded the data into the initial themes, which were then further refined to four key themes. In discussing the results, using quantitative descriptions to describe qualitative data was deemed inappropriate, therefore the frequency of a response is indicated by such terms as “all,” “most,” “many,” “some,” “a few,” or “one.” Two researchers from different disciplines developed the study (RM and AS) and analyzed the data (RM and SH). However, a total of 10 interviewers were involved in the data collection, due to the interviews being conducted in participants’ own languages. The use of multiple interviewers and coders assured that the findings are not a result of personal bias or leading questions and that the analysis is grounded in the data.

## Results

In total, in-depth interviews were conducted with 36 carers from the 11 countries (Table 1). All except four of the carers interviewed were the spouse of the person with PD. The others were carers to a parent or friend (two daughters, one son and one neighbour). The average age of the carers was 61 years, with the youngest aged 29 and oldest aged 82 years. 67% were female. Years since the person with PD they were caring for had been diagnosed ranged from two to 31 years.

**Table 1.** Participant Details by Country.

Country	Number of participants	Male: Female ratio	Current age at time of interview (average)/ range (years old)	Disease duration of people with PD (average number of years)/range (number of years)
Denmark	5	2:3	68/59-74	12.8/4-25
France	3	1:2	66.5/56-77	20.5/10-31
Germany	4	2:2	46.25/29-77	5/2-8
Hungary	3	2:1	69/64-77	6/2-10
Ireland	3	1:2	58/50-62	12/10-15
Italy	3	0:3	49/36-64	9.6/9-10
Netherlands	3	1:2	58.6/56-63	11/5-15
Slovenia	3	1:2	55.6/29-69	8/6-10
Spain	3	0:3	57.6/50-63	19/4-27
Sweden	3	1:2	74.6/70-82	15.6/10-20
UK	3	1:2	71/67-76	5.6/2-9

Similar themes and findings were identified across all 11 countries included in the study; the results are therefore presented combined for all countries.

### First Signs and Reaction to Diagnosis

Many of the carers reported they were the ones that had first noticed the symptoms and had subsequently encouraged their partner to seek help (more so if the PwPs was male).

“*I had had enough of his symptoms, being that we went everywhere together […] and all I heard was his shuffling his feet. And I told him one morning that I’ve had enough, it’s time to go and see the doctor.” (Carer, Hungary)*

*“I knew there was something wrong, maybe before he did, or before he admitted it maybe. He would just stand there, in the middle of a shop looking lost….” (Carer, Sweden)*

Around half of the carers attended the initial appointment with the person with Parkinson’s Disease (PwPs). On hearing the diagnosis, the main reaction was shock for the carers. However, many of the carers felt they needed to “*remain strong*”. They believed this was the best way to support the PwPs and the most effective way to “*move on*”.

*“I wanted to cry when I heard the news, but I looked at my wife and she…she was in bits. We have young children and I knew, I had to be the one to hold it all together.” (Carer, Ireland)*

### Carers Informational Needs

The informational needs differed as the disease progressed.

#### Information Needs at Time of Diagnosis

Information given at the time of diagnosis helped the carers to remain strong and also to “*calm down*” (recover from the initial shock).

*“When the diagnosis was given, the whole family was present – first you just stand, and can’t really realise it. Then you read a bit and calm down. You know it’s not a deathly disease and it’s developing slowly.” (Carer, Slovenia)*

The information given at the time of diagnosis to both the PwPs and the carer, usually came from the doctor making the diagnosis (or on a few occasions from a nurse assisting the doctor). This information was vital as most of the carers did not know anything about Parkinson’s or only knew about the tremor symptom, frequently due to knowledge of a well-known actor and boxer with the disease.

*“None of my other family members have Parkinson’s disease, it is not running in our family so I never suspected it. Sure, at first a bit of a shock, but I have never seen Parkinson’s as a threat, look at Michael J Fox, he is still alive and kicking.” (Carer, Germany)*

#### Use of the Internet/Websites

When given little information during the diagnostic appointment, or if the carer was not present at the appointment where the diagnosis was made, then the Internet was used to gain further information. However, this could cause great anxiety due to some of the information available online.

*“There was a lack of sensitivity by the neurologist when the diagnosis was given and no information was provided. Then I started researching it on the Internet. I was shocked as I did not realise it was such a distressing disease.” (Carer, France)*

#### Type of Information Needed

It was stressed by many of the carers that their informational needs differed from that of the PwPs. However, they believed that healthcare professionals did not always understand this.

*“Carers should know they have different needs and different questions about Parkinson’s. Health care professionals should not only focus on the patient but also on those things.” (Carer, Netherlands)*

Carers felt that, most importantly, they needed information about how to manage the medication. This was because managing medication was often their role, in particular as the disease progressed.

*“Once, I got very angry, because he had not properly arranged his medication before the holidays. I organize them now and remind him constantly. I feel like I am nagging but if I don’t…he just forgets.” (Carer, Netherlands)*

#### Specific Information Needed on Treatment Side Effects and Symptoms

In relation to information around medications, the carers wanted to be told about the side effects and what they should do/who they should contact if problems arose. The carers also wanted information on how to deal with certain symptoms, in particular the nonmotor symptoms, such as the behavioural issues and mental health problems that can occur.

*“We should get information on how to behave with the patient, because I tell you, sometimes I run out of patience. We had some problems with that and you don’t know who to turn to.” (Carer, Slovenia)*

#### Using the Information Gathered to Improve Treatment Outcomes

Many of the carers felt that they needed to be present at all appointments in case they needed to be an advocate for the PwPs They often used the information they gathered from the Internet to request different medications or demand referrals to other healthcare professionals (for example, to a physiotherapist).

*“My part of the care, because [partner’s name] was never sick, I had to go and fight his corner to get his meds reviewed.” (Carer, Ireland)*

### Support Needed During the Later Stages of the Disease

As the disease progressed, this added to the burden of care for the carers, in particular if they lived with the PwPs. The carers in this study articulated how it affected their quality of life in a number of ways.

#### Feelings of Isolation

Many of the carers felt increasingly isolated as the disease progressed, feeling both socially and emotionally isolated. For most of the carers it was due to their anxiety at leaving the PwPs alone in case of a fall or them forgetting to take their medication without them there.

*“For me it has changed my life totally. The first couple of months were not too bad as we could still get out and about freely but as the years have gone on… we are severely housebound.” (Carer, Ireland)*

#### The Need for ‘me Time’

Many of the carers, particularly those who cared for a PwPs in the later stages of the disease, talked about the need for time to themselves, even if it was just for a few hours a week to go to the shops, or sit in a café and read a book.

*“Two kinds of support could be done to support carers. First getting help with someone else to take care of him, so I even have some free hours to myself. And the other some kind of that would help me how to understand certain situations. For instance, he can’t eat, and for every meal there is a fight between us.” (Carer, Italy)*

#### Fear of Burdening Others

Carers often felt that they did not want to pass their burden of care onto other family members, in particular their children as they now had *“lives of their own”*. Many of the carers mentioned how difficult the diagnosis had been for their children, and the desire to “*protect*” their children from “*the reality of day-to-day life*”. Carers often felt that their own children had very busy lives (working, looking after their children, etc.) and that they did not have time to help out often.

### Existing Support Services for Carers

#### Support Groups

To try and overcome the feelings of isolation and to gain support, many of the carers talked about attending groups run by national Parkinson’s Associations. The reviews of these groups were mixed; whilst some of the carers gained great support from the groups, others attended mainly to gather information.

*“The doctor never gave me any specific information for my role as a carer, but I learnt through experience and from meetings with organised self-help groups for carers.” (Carer, Italy)*

At the support groups, some of the carers felt that they were not able to express their concerns and opinions freely, especially if they would be seen as a criticism of the PwPs that they cared for.

To try and address this issue, a few carers started attending nonspecific support groups (groups which were for all carers, and not just carers of PwPs). However, they usually left after a couple of sessions, as they were too general and again, they did not feel as if people understood the problems they were facing. Or that they felt guilty for “*moaning*” when some of the people attending had “*far greater*” issues (for example, caring for a severely disabled child which was seen by the carers interviewed as being far worst). Where the national Parkinson’s associations ran carer specific groups, this was greatly appreciated by the carers.

#### Use of Helplines

Where helplines were available, or when carers could phone and talk to a dedicated Parkinson’s disease nurse specialist, these were greatly appreciated and frequently used. The services were mainly used to ask questions about medications/side effects. However, they were also used by the carers to alleviate any worries they had as the disease progressed and gain advice on how to cope with certain symptoms.

## Discussion

This study, in which we conducted qualitative one-to-one interviews with 36 carers of PwPs, provides insights into the experience of carers of PwP from the time of diagnosis and as the disease progresses. The results are entirely focussed on carers own perspective and consider the range of experiences related to caring for someone with Parkinson’s across different European health systems.

Despite the cultural differences, the emerging themes were similar across the countries, with the carers’ expressing that they were playing an important role with responsibility for helping the PwP deal with and manage their Parkinson’s, the medication and its impact on their lives. However, other studies have shown that cultural values can impact on the experience of caring. Cultural values can affect the experience of caregiving as the updated sociocultural stress and coping model acknowledges.^8^ Specifically, values that permeate through families, communities and wider society are thought to influence the type of coping mechanisms employed by carers (i.e., social support, styles of coping). Research has illustrated how perceived strain in caregivers can be mitigated by cultural values, with evidence suggesting that communities who view caring for a family member as an important responsibility, and a role to ‘take pride’ in, describe these duties as less burdensome.^9^ A finding further supported by recent research that examined how caregiving was discussed in the media across 20 countries. Evidence from this study suggests that positive perceptions of caregiving were more evident in cultures that view these responsibilities as virtuous (Ng, et al., 2021).^(10)^

Even at the time of diagnosis, carers often felt that they had a role in helping obtain a diagnosis and then in dealing with the impact on the PwP on the family. Our previous qualitative study found that the diagnosis of PD was often a shock for many PwP.^7^ The results of this study in carers suggest that the diagnosis is often also experienced as a complete shock for their carers. Carers additionally frequently felt an added burden of having to “stay strong” and help the whole family “move on” and continue daily life in spite of the diagnosis. To help carers manage the stress and anxiety of the PD diagnosis and their new role as a carer, psychoeducational interventions and cognitive behavioural therapy may be beneficial to help them make the adjustment and increase coping skills.^1,11^

We also found that many carers felt their informational needs were different from those of the PwP and that this was not always appreciated by health professionals. This is similar to the situation in other conditions where a lack of tailored information specifically for carers has been reported.^12^ The main source of support and information in Europe at present is Parkinson’s Europe (formerly EPDA) and country specific organisations such as Parkinson's UK. The organisations all have their own websites and some have support telephone lines and run carer meetings. However, the availability of support and information can vary across countries and within countries.

Carers of PwP felt that they have an important role in medication management, access to health care, self-management and future care planning, where they were often lacking sufficient information. In order to gather information, many turned to the internet, which was sometimes frightening. On the other hand, information received from reliable sources, such as the health professionals or Parkinson’s organisations helped to alleviate fears and support them. As there is often little knowledge on PD at the time of diagnosis, this is an important time for carers to be provided with information. The informational needs of carers can also change as the disease progresses and new challenges arise and information appropriate to the challenges should be provided.

Disease duration of the people with Parkinson’s Disease that were being cared for ranged from 2 to 27 years and the study therefore included carers of patients with disease durations ranging from relatively recent diagnosis and more than two decades. As the disease progressed, the burden on the carers increased. Carers of those in the later stages of the disease often reported feeling isolated and not having any time for themselves. Spousal carers frequently felt that they could not share this burden with other younger family members, but that they would appreciate a support network of other carers caring for someone with PD. To help carers feel conformable speaking about their worries and concerns, and personal situations, support groups should be established for carers which are either dealing with different progressive neurological illnesses or PD specific support groups. Other studies have found specialised groups to be a helpful and supportive environment for carers^13^.

## Strengths and limitations of the Study

This paper reports on a qualitative study which followed the principles of Grounded Theory to try and enhance the validity of the findings. However, due to the recruitment of participants, which was done through national Parkinson’s Associations, a selection bias cannot be excluded. Although the research team did recruit some participants who were not actively involved in their national Association, these were the minority. This has potentially led to a more informed and active sample of carers . The study did not identify differing issues or themes between younger and older carers or between male and female carers, which may have been due to the small sample size of this qualitative study. This also precluded meaningful comparison between countries as the number recruited for each countries was relatively small.

## Conclusion and Implications for Practice and Future Research

Coping with PD is a complex process for both PwPs and their carers. The involvement of carers should be addressed more actively in the management of Parkinson’s, if the PwP indicates this is their wish. Family carers often play an important role from the time of diagnosis, and support self-management, and they may require different information than the PwP wishes to receive at a particular time. This is particularly important for medication management but also when planning future arrangement for the later stages, which also have a profound effect on carers’ lives.
